# Hospital Laundries, Metropolitan and Provincial

**Published:** 1912-11-16

**Authors:** 


### PRACTICAL DEPARTMENTS.

### Hospital Laundries, Metropolitan and Provincial.

The plan pursued by various hospitals in providing for their washing shows considerable variations. The chief are: (1) The case of hospitals where washing is done entirely on the premises; (2) those hospitals which send their washing out, or have it done under their own control and direction in an establishment entirely separate and distinct from the hospital. Under the Uniform System the hospitals in the first type, which includes also all institutions where the washing is not done entirely apart from the establishment, have to furnish a return, which should be published in the annual report, of its expenditure on washing or any part of such washing done on the hospital premises. In the case of those hospitals which send their washing out, or do it themselves in an establishment entirely separate and distinct from the hospital, the total cost involved has to be entered under "Domestic," "Washing" in the income and expenditure account for each year. These alternative systems sometimes cause much discussion as to their respective merits, and more as to the right way of treating the figures relating to them under the Uniform System. We have, therefore, thought it would be a matter of great interest to publish in this Special Number dealing with laundries an account of the laundry and its working at the London Hospital, Whitechapel, which does its own washing on the premises, and also an account of the Middlesex Hospital Laundry at Hendon, where the laundry constitutes an establishment entirely separate and distinct from the hospital proper; these illustrate the cost of working of the alternative systems.

We have decided to offer no comment at the present time nor to indulge in any comparisons as to the results attained by the two systems, believing that it will be a better service to hospital workers and authorities everywhere to give the two systems as they are at present working in connection with two important Metropolitan hospitals, so that all interested may study the plan pursued and the figures relating to each of them for themselves.

We take this opportunity of expressing our indebtedness to the authorities of the London and the Middlesex Hospitals for their great courtesy and kindness in enabling us to publish the following particulars of the important laundries which are under their control. As will be seen, the Middlesex article is especially full of helpful details about the work as it is actually carried out.

